# Impact of immunogenicity on clinical efficacy and toxicity profile of biologic agents used for treatment of inflammatory arthritis in children compared to adults

**DOI:** 10.1177/1759720X211002685

**Published:** 2021-06-16

**Authors:** Chinar R. Parikh, Jaya K. Ponnampalam, George Seligmann, Leda Coelewij, Ines Pineda-Torra, Elizabeth C. Jury, Coziana Ciurtin

**Affiliations:** Centre for Adolescent Rheumatology versus Arthritis, University College London, London, UK; Medical School, University College London, London, UK; Centre for Adolescent Rheumatology versus Arthritis, University College London, London, UK; Medical School, University College London, London, UK; Centre for Adolescent Rheumatology versus Arthritis, University College London, London, UK; Medical School, University College London, London, UK; Centre for Rheumatology Research, University College London, London, UK; Centre for Cardiometabolic and Vascular Science, University College London, London, UK; Centre for Rheumatology Research, University College London, London, UK; Centre for Adolescent Rheumatology Versus Arthritis, University College London, 3rd Floor Central, 250 Euston Road, London NW1 2PG, UK; Centre for Adolescent Rheumatology versus Arthritis, University College London, London, UK

**Keywords:** anti-drug antibodies, drug levels, inflammatory arthritis, immunogenicity

## Abstract

The treatment of inflammatory arthritis has been revolutionised by the introduction of biologic treatments. Many biologic agents are currently licensed for use in both paediatric and adult patients with inflammatory arthritis and contribute to improved disease outcomes compared with the pre-biologic era. However, immunogenicity to biologic agents, characterised by an immune reaction leading to the production of anti-drug antibodies (ADAs), can negatively impact the therapeutic efficacy of biologic drugs and induce side effects to treatment. This review explores for the first time the impact of immunogenicity against all licensed biologic treatments currently used in inflammatory arthritis across age, and will examine any significant differences between ADA prevalence, titres and timing of development, as well as ADA impact on therapeutic drug levels, clinical efficacy and side effects between paediatric and adult patients. In addition, we will investigate factors associated with differences in immunogenicity across biologic agents used in inflammatory arthritis, and their potential therapeutic implications.

## Introduction

The discovery and clinical use of biologic treatments in the management of inflammatory arthritis in children and adults has been associated with significant clinical benefits, as well as advances in understanding the pathogenesis of different types of inflammatory arthritis. Immunogenicity to biologic treatments is an unwanted immune reaction against a therapeutic antigen. This immune reaction generates anti-drug-antibodies (ADAs), which could counteract the therapeutic effects of the biologic treatment and, in rare cases, induce adverse reactions.^1,2^

It has become increasingly recognised that biologic treatment duration, mode, rate and route of administration, and more specifically, the type of biologic therapeutic [e.g. monoclonal antibodies (mAbs) *versus* recombinant fusion proteins] are all factors that influence the risk of immunogenicity.^3^ In addition, individual patient factors, such as genetic background,^4^ disease type,^5^ and concomitant use of disease modifying anti-rheumatic drugs (DMARDs),^6^ all contribute differentially to the formation of ADAs. Recent research has been focused on highlighting the genetic risk for developing ADAs: e.g. HLA-DRB1*15 was associated with increased the risk for developing high ADA levels to interferon (IFN)β-1a treatment in multiple sclerosis, while HLA-DQA1*05 decreased this risk,^7^ and HLA-DQA1*05 was associated with increased ADA prevalence across various biologics and autoimmune diseases.^8^ Other factors such as smoking and infections are also associated with increased risk,^8,9^ whereas concomitant use of antibiotics and immunosuppressant medication are associated with decreased immunogenicity risk.^8^ In addition, the manufacturing process of various biologic agents, in particular, their contamination with low-level host proteins, is a major contributor to immunogenicity.^10^

Therapeutic drug monitoring and immunogenicity testing comprise measurement of trough drug levels and ADAs. The most widely used ADA detection methods are bridging enzyme-linked immunosorbent assay (ELISA; which use labelled therapeutic mAbs) and radioimmunoassay (RIA), while other new methods such as competitive displacement and tandem mass spectrometry have also been proposed.^11^ Currently, most mAbs on the market are humanised or fully human; however, they still carry immunogenic risk. This could be attributed to anti-idiotype reactivity, which is a common reaction of the immune system to the appearance of any novel antibody.^12^

The molecular mechanisms leading to generation of ADA are not completely elucidated and a detailed discussion of immune mechanisms is beyond the scope of this review (for a recent review see^13^). One basis for ADA generation involves the capacity of the human immune system to recognise ‘non-self’. Since the first therapeutic mAbs of murine origin were developed, further efforts have now been made to improve their performance and decrease their immunogenicity. The continuous advancement in recombinant deoxyribonucleic acid (DNA) technologies has led to the development of chimeric (fused human–murine mAbs) and humanised mAbs. Chimeric antibodies were developed by replacing the constant region of murine mAbs with human components and the humanised mAbs are constituted entirely of human sequences, with the exception of the complementarity determining regions of the variable regions which are of mouse-sequence origin. Subsequently, the advanced antibody engineering achieved the production of fully human antibodies where antigen specificity has been selected either *in vivo* in genetically modified mice or by antibody engineering processes combined with screening.^14^ Many factors contribute to differences in immunogenicity, from biopharmaceutical properties related to downstream processing and drug formulation^15^ to patient individual characteristics, including the antigen burden which correlates with their disease activity.^16^

Both ELISAs and RIAs detect only free circulating ADAs; therefore, they can be associated with false negative results in the context of presence of ADA-immune complexes which are detectable only if they exceed in concentration the circulating drug levels.^17,18^ In one study, ELISA was more sensitive in detecting ADA when present in high titres than RIA, while in patients with ADA detected by RIA but not by enzyme-linked immunosorbent assay, only the drug levels were significantly associated with treatment response to adalimumab.^19^ Interestingly, measuring drug levels and drug clearance alone is also shown to be a reliable predictor for ADA in RA and juvenile idiopathic arthritis (JIA) patients.^20,21^ Several studies concluded that although ADAs were not independently associated with treatment response, they may be helpful in determining the cause of low drug levels and guide therapeutic decisions.^22,23^

The presence of ADAs may be associated with reduced clinical efficacy through two main mechanisms. ADAs that compete with the cytokine binding site (the Fab fragment of the therapeutic agent) have neutralising properties as they block the pharmacological function of the drug. ADAs directed against the Fc fragment (more frequently targeting the junction between Fc and Fab) lead to formation of immune complexes associated with enhanced drug clearance that may also influence the clinical response to biologic treatment through leading to sub-optimal (sub-therapeutic) drug levels.^24^ Therefore, based on their specificity ADA can be grouped as neutralising (when they target the antigen binding sites of the therapeutic drug) or non-neutralising (when they recognise epitopes away from the drug-binding site, therefore not directly impairing the efficacy of the drug).^3^

Here, we review the evidence of impact of ADAs against various biologic therapeutics used for treatment of inflammatory arthritis in adults and children, as there are no previous reports investigating immunogenicity across age. This review focuses on depicting differences between ADA prevalence, titres and timing of development, as well as impact on therapeutic drug levels, clinical efficacy and side effects in children compared with adults with inflammatory arthritis. Where data are available, we will also investigate the clinical predictors for ADA development, as well as the influence of additional DMARD therapy on ADA development and biologic drug retention.

### Neutralising ADAs against mAbs targeting TNF-α were more prevalent than ADA against fusion proteins (etanercept and biosimilars) while the kinetic of ADA generation varied across anti-TNF-α agents in adult and paediatric inflammatory arthritis studies

Many studies have reported the presence of ADAs against anti-tumour-necrosis-factor-alpha (anti-TNF-α) inhibitors used to treat different types of inflammatory arthritis, including etanercept [fusion protein of the extracellular ligand-binding portion of the human 75KD p75 TNF receptor (TNFR) linked to the Fc portion of human immunoglobulin G1 (IgG1)], adalimumab (fully human mAb), certolizumab (humanised antibody Fab’ fragment), golimumab (human IgG1κ monoclonal antibody) or infliximab (a chimeric mAb; Table 1). The general observation is that ADAs against etanercept have a lower prevalence compared with ADAs against adalimumab or infliximab.^25^ Furthermore, comparative studies show that ADAs to human/humanised (adalimumab, certolizumab, golimumab) and chimeric (infliximab) anti-TNF-α therapeutic mAbs are largely neutralising,^26^ while the ADAs against etanercept are predominantly non-neutralising.^27^

**Table 1.** Impact of ADAs on disease outcomes in children and adults with inflammatory arthritis treated with anti TNF-α agents.

Author(s) and reference number	Country Type of study (including meta-analyses) Number of patients treated with a certain biologic	Type of inflammatory arthritis Age range or mean age (years)	Disease duration Range or mean ± SD (years)	Prevalence of ADAs Impact of additional DMARD therapy on ADA prevalence	Impact on clinical efficacy	Impact on side effects to biologic therapy
Adalimumab and biosimilars						
Strand *et al*.^28^	Systematic review RA *n* = 1282 PsA *n* = 59 JIA *n* = 23 AS = 204	RA (35–64) PsA (43–55) JIA (3–14.2) AS (30–48)	RA: 1–34 PsA: 5–21 JIA: 1–5 AS: 4–15	RA 0–51%; PsA 0–54% JIA 6–33%; AS 8–39% Concomitant use of MTX, AZA, leflunomide or MMF was associated with lower rates of ADA in RA, JIA, AS	ADA was associated with less improvement of disease activity for RA, PsA and AS. A higher proportion of ADA+ve JIA patients experienced loss of response than ADA−ve patients (no *p* value reported)	Adverse events occurred more frequently in ADA+ve patients compared to ADA−ve (27% *versus* 15%, no *p* value reported)
Doeleman *et al*.^29^	Systematic review and meta-analysis *n* = 355	JIA 10.5	3.45	Pooled prevalence of 21.5% (95% CI = 14.1 to −29.8) Addition of MTX reduced the risk of ADA development by 67% (RR 0.33)	Increased median disease activity score in patients with ADA was found (no *p* value reported)	No association with adverse events generally was found, but in patients with JIA-associated uveitis, ADA were associated with a significantly higher severity of uveitis (no *p* value reported)
Marino *et al*.^30^	Italy Prospective observational study *n* = 27	JIA Age at inclusion 9.5 ± 3.32 ADA+ve 11.15 ± 3.11 ADA−ve 8.52 ± 3.12	4.79 ± 3.04	Overall prevalence 37% 31% *versus* 45% in MTX+ve *versus* MTX−ve groups No impact of MTX treatment duration on ADA development was found: 22.9 months (MTX+ve group) *versus* 17.8 months (MTX−ve group)	ADA+ve patients experienced more relapses, *p* < 0.017. 30% of ADA+ve patients were in clinical remission, compared to 41.2% of ADA– patients, *p* = 0.56	No infusion reactions or side effects were found
Maid *et al*.^31^	Argentina Cross-sectional study *n* = 52	RA 56.5 (13.3)	10.8 ± 8.5	36.5% 36% of MTX+ve patients and 38% of MTX−ve patients tested positive for ADA	ADA−ve patients had a tendency towards better clinical outcomes than those who were ADA+ve −39.4% of ADA−ve patients achieved a HAQ-DI score < 0.5, compared with only 31.6% of ADA+ve patients (comparative statistics were not performed)	Injection site reactions were reported by 6.3% in the ADA−ve group and 4.3% in the ADA+ve group (no *p* value reported) (combined data for adalimumab, infliximab and etanercept)
Balsa *et al*.^32^	Spain Cross-sectional, observational study *n* = 217	RA and SpA RA = 56.3 (12.1) SpA = 47.9 (11.5)	RA = 13.9 ± 8.7 SpA = 12.5 ± 10.2	RA: 25.5% SpA: 32.7% No significant difference between the two patient groups (*p* = 0.221) Lower proportion of patients receiving concomitant DMARDs (24.1% *versus* 36.9% were ADA+ve, *p* = 0.037)	82.5% ADA+ve patients had no detectable drug levels in the serum; only one ADA+ve patient reported drug concentrations within the normal range; no *p* value reported	Data not available
Quistrebert *et al*.^9^	European retrospective multi-cohort analysis *n* = 240	RA 50.3	2.18	19.2% 96.6% of patients were MTX+ve, but study was not powered to analyse the effects	ADA positivity was significantly associated with a lower probability of a good clinical response based on 278 clinical observations from 215 patients (hazard ratio = 0.58, 95% CI 0.39–0.86)	Data not available
Verstegen *et al*.^33^	Systematic review *n* = 103	JIA 10.6	Data not available	6.7–37% Concomitant treatment with MTX showed a protective effect against ADA development for patients treated with adalimumab and infliximab	ADAs to adalimumab were associated to impaired clinical outcome (no comparative statistics performed)	Data not available
Skrabl-Baumgartner *et al*.^34^	Austria Prospective observational study *n* = 20	JIA 9.9 ± 4.2	JIA data not available Duration of JIA-associated uveitis 3.5 ± 3.5	45% (including permanent and transient ADAs) Concomitant use of DMARDs significantly lower in group with permanent ADA+ve (2/7) *versus* ADA−ve (10/11); *p* < 0.05	7/8 who had a loss of response had permanent ADAs Transient ADAs were not associated with a diminished response (no comparative statistics performed)	No severe adverse reactions were found
Moots *et al*.^27^	Multinational non-interventional study *n* = 199	RA 54.3 ± 12.95	Symptom duration 9.3 ± 8.43	RA 31.2%	Significant differences between patients with and without detectable ADAs were observed in ESR (*p* = 0.008) and CRP (*p* = 0.0011) When data for all three TNF inhibitors were pooled, a greater proportion of patients without detectable ADAs (226/484; 46.7%) than those with detectable ADAs (29/94; 30.9%) were in remission (*p* = 0.0046)	No differences in safety outcomes were reported
Infliximab and biosimilars						
Strand *et al*.^28^	Systematic review RA *n* = 1412 PsA *n* = 173 JIA *n* = not available AS *n* = 163	RA (35–64) PsA (43–55) JIA (3–14.2) AS (30–48)	RA: 1–34 PsA: 5–21 JIA: 1–5 AS: 4–15	RA 8–62%; PsA 15–33%, JIA 26–42%; AS 6.1–6.9%; Concomitant use of MTX, AZA, leflunomide or MMF was associated with lower rates of ADA in RA	ADA+ve patients showed less improvement in disease activity and were less likely to achieve clinical responses (RA, PsA, AS; no comparative statistics performed)	Increased risk of treatment discontinuation due to adverse events and higher rates of infusion reactions were reported in ADA+ve patients (no comparative statistics performed)
Maid *et al*.^31^	Argentina Cross-sectional study *n* = 13	RA 55.5 (10.6)	13.1 ± 8.5	30.8% 22.2% of MTX+ve and 50% of MTX−ve patients had ADAs	ADA−ve patients had a tendency towards better clinical outcomes than those who were ADA+ve: no comparative statistics were performed due to low numbers.	Injection-site reactions were reported by 6.3% in the ADA−ve and 4.3% in the ADA+ve group (no *p* value reported; combined data for adalimumab, infliximab and etanercept)
Balsa *et al*.^32^	Spain Cross-sectional, observational study *n* = 188	RA and SpA RA = 56.3 (12.1) SpA = 47.9 (11.5)	RA = 13.9 ± 8.7 SpA = 12.5 ± 10.2	RA: 21.1% SpA: 31.3% No significant difference between the two patient groups (*p* = 0.114) Concomitant use of DMARDs associated with lower ADAs: ADA−ve 29/130 (22.3%) *versus* 22/58 ADA+ve (37.9%; *p* = 0.021)	78.4% ADA+ve patients had no detectable drug in the serum. Only one ADA+ve patient reported drug concentrations within the normal range; no *p* value reported	Data not available
Quistrebert *et al*.^9^	European retrospective multi-cohort analysis *n* = 126	RA 50.6	2.65	RA 29.4% ADAs were detected more frequently in infliximab-treated patients (29.4%) than in adalimumab-treated patients (19.2%)	ADA positivity was significantly associated with a lower probability of a good clinical response based on 149 clinical observations from 125 patients (hazard ratio = 0.61, 95% CI 0.32–0.76)	Data not available
Ruperto *et al*.^35^	Multicentre RCT *n* = 122	JIA 11.2	3.9	25.5%	Data not available	Infusion reactions were observed in 58% of ADA+ve patients compared with 19% of ADA−ve patients Serious infusion reactions additionally occurred in 20% of ADA+ve patients, compared with 0% of ADA−ve patients No comparative statistics performed
Ruperto *et al*.^36^	Multicentre open-label extension study *n* = 78	JIA Data not available	Data not available	37% (+32% inconclusive)	Data not available	32% patients had ⩾1 infusion-related reaction, with a higher occurrence among patients who were ADA+ve [15/26 (58%) ADA+ve patients had infusion-related reactions] No comparative statistics performed
Moots *et al*.^27^	Multicentre noninterventional study *n* = 196	RA 60.7 ± 13.01	Symptom duration 10.0 ± 10.11	RA 17.4%	95/184 (51.6%) were in low disease activity, of which 14/32 (43.8%) had detectable ADAs and 81/152 (53.3%) had no detectable ADAs (*p* = 0.3387) Significant differences between patients with and without detectable ADA were observed in ESR (*p* < 0.0001) and CRP (*p* = 0.0001)	No significant correlation between adverse events and ADAs was found
Etanercept and biosimilars						
Strand *et al*.^28^	Systematic review RA *N* = 589 PsA, JIA, AS *n* = not available	RA (35–64) PsA (43–55) JIA (3–14.2) AS (30–48)	RA: 1–34 PsA: 5–21 JIA: 1–5 AS: 4–15	RA 0–13% PsA 0% JIA 0–6% AS 0%	Data not available	Data not available
Balsa *et al*.^32^	Spain Cross-sectional, observational study *n* = 165	RA and SpA RA = 56.3 (12.1) SpA = 47.9 (11.5)	RA = 13.9 ± 8.7 SpA = 12.5 ± 10.2	RA: 0% SpA: 0%	Data not available	Data not available
Doeleman *et al*.^29^	Systematic review and meta-analysis *n* = 268	JIA 11.8	4.7	Pooled prevalence 8.5% (95% CI = 0.5 to −23.2)	No reported association between treatment failure and the presence of non-neutralizing ADAs	No association between adverse events and ADAs was observed
Maid *et al*.^31^	Argentina Cross-sectional study *n* = 54	RA 54.5 (13.6)	12.5 ± 10.1	0%	Data not available	Data not available
Bader-Meunier *et al*.^37^	France Prospective multicentre study *n* = 126	JIA 10.5 (2–17)	4.62 (0.16–16.3)	15.7% at baseline 33% after 366 (302–712) days of treatment	ADA levels not significantly different between responders and non-responders (7.22 ± 3.60 *versus* 6.47 ± 3.98 ng/ml), No significant difference with concomitant MTX *p* values <0.05 were considered significant	No severe adverse events occurred
Moots *et al*.^27^	Multicentre non-interventional study *n* = 200	RA 56.5 ± 13.37	Symptom duration 0.8 ± 10.67	0%	No patients developed ADAs on etanercept)	Data not available
Constantin *et al*.^38^	Multicentre prospective open-label study *n* = 127	JIA 8.6 ± 4.6 ERA 14.5 ± 1.6 JPsA 14.5 ± 2.0	JIA 31.6 ± 31.7 months ERA 23.0 ± 19.8 months JPsA 21.8 ± 20.2 months	JIA 18.3%, ERA 23.7%, JPsA 20.5%, combined: 20.7% None of the ADA+ve patients had neutralising antibodies	No significant changes in effectiveness in patients who were ADA+ve was found	No safety concerns in patients who were ADA+ve were reported
Golimumab						
Strand *et al*.^28^	Systematic review RA *n* = 1249 PsA, JIA and AS *n* = not available	RA (35–64) PsA (43–55) JIA (3–14.2) AS (30–48)	RA: 1–34 PsA: 5–21 JIA: 1–5 AS: 4–15	RA: 2–10% PsA: 6% AS: 0–6.4% Concomitant use of MTX, AZA, leflunomide or MMF was associated with lower rates of ADAs in RA, PsA and AS	ADA+ve RA patients showed less improvement in disease activity and were less likely to achieve clinical responses (no comparative statistics performed)	Data not available
Brunner *et al*.^39^	Multicentre withdrawal RCT *n* = 154	JIA 11.1 ± 4.5	Disease duration not available	46.8% (72/154)	ADAs did not appear to have a substantial impact on clinical efficacy	ADAs were not associated with injection-site reactions, disease flares or adverse events
Leu *et al*.^40^	Samples from 3 RCTs	RA PsA AS	Data not available	RA: 24.9% PsA: 39.9% AS: 30.3%	No effect of ADA on clinical response was found	Injection-site reactions were not affected by ADAs
Kneepkens *et al*.^41^	The Netherlands Prospective observational cohort study *n* = 37	RA	Data not available	8.1%	3 patients out of 37 (8.1%) were ADA+ve at 52 weeks and all 3 discontinued golimumab prematurely due to inefficacy	Data not available
Certolizumab						
Strand *et al*.^28^	Systematic review RA *n* = 358 PsA, JIA and AS *n* = not available	RA (35–64) PsA (43–55) JIA (3–14.2) AS (30–48)	RA: 1–34 PsA: 5–21 JIA: 1–5 AS: 4–15	RA 2.8–37% Concomitant use of MTX, AZA, leflunomide or MMF was associated with lower rates of ADA	Data not available	Data not available
Gehin *et al*.^42^	Norway Longitudinal observational study *n* = 116	RA, AS, PsA and other inflammatory joint disease 42	2.6 0.6–14.1	Prevalence 6.1% (19/310 patients: 6 AS, 5 RA, 4 PsA and 4 other IJD) Among RA patients, 80% of ADA+ve patients had concomitant synthetic DMARDs (mostly MTX) *versus* 73% of ADA−ve patients	9% ADA+ve patients were responders at 3 months *versus* 55% of ADA−ve patients No *p* value reported	Data not available 8 patients experienced one or more injection-site reactions, all of which were ADA−ve at 3 months
Jani *et al*.^43^	The Netherlands Prospective observation cohort study *n* = 115	RA 58.0 ADA+ve 57.3 ADA−ve 58.5	7.0 3.3–14.4 ADA+ve 8.3 ADA−ve 6.0	37%	No correlation between ADA+ve and EULAR response was found (*p* = 0.18)	Data not available

+ ve, positive; −ve, negative; ADA, anti-drug antibody; AS, ankylosing spondylitis; AZA, azathioprine; CI, confidence interval; CRP, C-reactive protein; DMARD, disease-modifying antirheumatic drug; ERA, enthesitis-related arthritis; ESR, erythrocyte sedimentation rate; EULAR, European League Against Rheumatism; HAQ-DI, Health Assessment Questionnaire Disease Index; IJD, inflammatory joint disease; JIA, juvenile idiopathic arthritis; JPsA, juvenile psoriatic arthritis; MMF, mycophenolate mofetil; MTX, methotrexate; *n*, number of patients treated with a certain biologic included in the study/systematic review; PsA, psoriatic arthritis; RA, rheumatoid arthritis; RCT, randomised control trial; SD, standard deviation.

In adults, the rates of ADA formation against infliximab range from 8% to 62% in rheumatoid arthritis (RA), 15% to 33% for psoriatic arthritis (PsA) and 6.1% to 69% for ankylosing spondylitis (AS;^28^ Table 1). ADAs against infliximab are also shown to be associated with lower serum biologic drug concentrations in adult inflammatory arthritis patients.^27,28,31,32,44–48^ There is a paucity of studies investigating the timing of development of ADA against various anti-TNF-α agents: evidence suggests that longer exposure to infliximab increases immunogenicity; for example, ADAs against infliximab in adults with RA occurred after the first 10 infusions (23.4 ± 2.4 weeks), while ADAs were detected in 25% of JIA patients after 52 weeks and in 37% at 204 weeks.^35,36,49^ The dose of biologic agent, as well as patients’ age, could influence immunogenicity: a higher incidence of ADAs was observed in patients treated with infliximab 3 mg/kg (38%), compared with 6 mg/kg (12%),^36^ while a significantly higher prevalence of ADAs was found in younger children (ADA-positive mean age 7.01 years *versus* ADA negative 9.88 years, *p* = 0.003).^29^

The prevalence of ADAs against adalimumab has high variability across different types of autoimmune diseases in adults^25,28,31,50–52^ and children with JIA^35^ (Table 1). The timing of adalimumab ADA development is controversial: in some adult studies ADA prevalence did not increase with treatment duration,^53,54^ while in other studies there was a significant increase, with ADA developing between 4.5 months and 12 months of treatment.^9,34,44,50,52,55^ Similarly, studies in JIA showed both trends: a significant increase of ADA with time^35^ or no correlation with treatment duration,^30^ suggesting that ongoing monitoring to establish their clinical relevance and impact on management is required.

Etanercept treatment was associated with a lower ADA rate than infliximab and adalimumab^25^ (Table 1), with the vast majority of adult studies reporting no detectable ADA^25,27,28,31,32,50,52,55^ This pinpoints that the chemical structure of the anti-TNF-α therapeutic agent (fusion protein *versus* mAb) is likely to be a key factor in inducing drug immunogenicity. When detected, ADAs against etanercept were found to be non-neutralising in both adult and paediatric studies.^28,35^ ADA prevalence increased with treatment duration with a corresponding decrease in etanercept drug levels over time in JIA.^37,38^

A highly sensitive ELISA test detected ADA against golimumab in 31.7% of patients with RA, PsA and AS in comparison with standard ELISA which detected ADA only in 4.1%,^40^ while their prevalence varied across adult studies (Table 1). The impact of ADA on serum golimumab concentrations was consistent in JIA and RA studies, whereby higher ADA titres were associated with lower drug concentrations.^28,39,41,56^ This was generally shown at ADA titres >1:1000 in JIA,^39^ and in adults, median peak titres ⩾100 were associated with undetectable or very low drug levels.^57^ Interestingly, in another study in PsA, which used a standard assay, the golimumab dose (50 mg *versus* 100 mg) did not appear to affect the ADA rates, which remained low for the whole duration of the study through to week 52 (4.9%).^58^

There are fewer studies investigating the presence of ADAs against certolizumab,^42,43^ although in both studies, ADAs were associated with lower drug levels (Table 2). A more recent study, however, reported that there was no significant correlation between ADA and certolizumab drug levels (*r* = −0.471, *p* = 0.122). There is evidence that ADAs were still detected at higher certolizumab concentrations of >10 mg/l.^59^ The majority of patients with ADA had detectable titres from week 16 onwards, and 65% remained ADA positive after 1 year of follow up.^59^ There are no studies in paediatric populations.

**Table 2.** Impact of ADAs on disease outcomes in children and adults with inflammatory arthritis treated with other biologic agents.

Author(s) and reference number	Country Type of study	Type of inflammatory arthritis *n* (F:M) Age (mean ± SD)	Disease duration		Prevalence of ADAs Impact of additional DMARD therapy on ADA prevalence	Impact on clinical efficacy	Impact on side effects
B-cell depletion (rituximab and biosimilars)							
Strand *et al*.^28^	Systematic review	RA Patient demographics n/a	Data not available		0–21%	Patients with ADAs *versus* RTX showed less improvement in disease activity and were less likely to achieve clinical responses in RA patients; no comparative statistics/meta-analysis performed	Higher rates of treatment-emergent adverse events (89% *versus* 68%) were reported in patients with RA who develop anti-RTX ADAs compared with those who did not
Thurlings *et al*.^60^	The Netherlands Open-label cohort study	RA *n* = 58 (F:M = 44:14)	Data not available		Data not available	Response to treatment and re-treatment measured by decrease in DAS28 and EULAR response was similar in ADA-positive and ADA-negative patients: *p* = 0.87 and *p* = 0.32 for the responses at 24 weeks after courses 1 and 2, respectively)	Data not available
Combier *et al*.^61^	France Retrospective cohort study	RA *n* = 124 (F:M = 97:27) Age (mean = 62; range 22–89) Other ARDS (including pSS, SLE, myositis) *n* = 75 (F:M = 59:16) Age (mean = 57; range 21–85)	RA 13 years (1–60) Other ARDS 10 years (1–28)		RA 2.4% Other ARDS 14.7%	No data available on ADA impact on clinical efficacy 14.29% were tested because of loss of efficacy, and 78.6% were tested because of adverse reactions No comparative statistics performed	78.57% of ADA+ve patients (48/62 tested) with RA and other ARDs had infusion reactions to second or subsequent RTX cycles
Co-stimulatory blockade (abatacept)							
Strand *et al*.^28^	Systematic review	RA (age 35–64) JIA (age 3–14.2) RA: *n* = 1993 JIA: *n* = not available	RA: 1–54 JIA: 1–5		RA 2%–20% JIA 2%–11% Suggested that IV therapy associated with less immunogenicity than SC	Data not available	Data not available
Doeleman *et al*.^29^	Systematic review and meta-analysis	JIA IV: *n* = 190 SC: *n* = 173 Mean age IV: 12.4 (3.0) SC: 13.0 (10.0–15.0)	IV: 4.4 (3.8) SC: 2.0 (0.0–4.0)		9.9% (pooled from 3 studies) (95% CI = 0.3–28.6)	No association between ADAs and treatment failure was found	No injection-site reactions experienced with SC and no adverse reactions for IV formulations were described
Hara *et al*.^62^	Japan Open label, multicentre single arm study	JIA IV *n* = 20 Mean age 10.5 years (5–16) 4–8 years: 40% 9–12 years: 35% 13–17 years: 25%	0.75 (0.2–11.9)		5% (IV only)	No association between immunogenicity and loss of efficacy was found No comparative statistics performed	No association with safety, adverse events or hypersensitivity was found
Brunner *et al*.^63^	International open label, multicentre study single-arm study	JIA: *n* = 219 2–5 years: *n* = 46, median age 4.0 (3.0–5.0) 6–17 years: *n* = 173, median age 13.0 (10.0–15.0)	2–5 years, 0.5 (0.0–1.0) 6–17 years 2.0 (0.0–4.0)		2.3% 6–17 years 8.7% 2–5 years (SC only)	No clinical significance of ADAs was found	No issues regarding safety were found
Lovell *et al*.^64^	Multicentre RCT	JIA *n* = 58 (active arm) *n* = 59 (placebo) Mean age 12.4 ± 2.9	3.8 ± 3.8		Whole abatacept molecule 3.4% (2/58) CTLA-4 region only 5.5% (9/58; IV only)	No loss of efficacy was found in the two patients with anti-abatacept antibodies to the whole molecules Of the 9 patients with ADA against the CTLA-4 region, 3 discontinued due to lack of efficacy (small sample size, so no comparative statistics performed)	No infusion reactions were experienced
Haggerty *et al*.^65^	Integrated analysis across multiple double-blind and open-label studies	RA *n* = 2237	Data not available		RA 2.1% ADA+ve with MTX 2.3% *versus* ADA+ve without MTX 1.4%: not significant	Patients who discontinued had a higher level of ADAs compared with those who did not discontinue (7.4% *versus* 2.6%); no comparative statistics performed	No adverse safety outcomes were described
IL-6 blockade (tocilizumab/sarilumab)							
Benucci *et al*.^66^	Italy Cohort study of tocilizumab	RA *n* = 126 (F:M = 110:16) Mean age: 59 ± 12 years Range: 26–83 years	Mean disease duration: 11 ± 5 years		0.79% (1/126 patients)	The occurrence of ADAs against Tocilizumab is very rare	Data not available
Sigaux *et al*.^67^	France Cohort study of tocilizumab	RA *n* = 40 (F:M = 32:8) Mean age: 56.5 ± 14 years	16 ± 11.7 months		3.2%	No association between ADA status and disease activity was found	
Burmester *et al*.^68^	Meta-analysis of phase III RCTs of Tocilizumab	RA TCZ-SC: *N* = 3099 TCZ-IV: *N* = 5875	Data not available		TCZ-SC: 1.5% TCZ-IV: 1.2%	No association with decreased clinical efficacy was found	No clear impact of ADA on safety and side effects was found
Yokota *et al*.^69^	Japan Phase II–III RCTs of tocilizumab	sJIA *n* = 67 (F:M = 38:29) Mean age: 8.3 ± 4.3 years	4.4 ± 3.5 years		7.5%	No decrease in clinical effectiveness was reported	4/5 patients with ADAs experienced mild to moderate infusion reactions
Burmester *et al*.^70^	Multicentre RCT of sarilumab	RA *n* = 184 (F:M = 157:27) Mean age: 50.9 ± 12.6 years	8.1 ± 8.1 years		7.1%	ADAs were not associated with a loss of efficacy	ADAs were not associated with hypersensitivity reactions
Wells *et al*.^71^	USA Open-label study of sarilumab	RA *n* = 132 (F:M = 106:26) Mean age: 52.4 ± 13.4 years	10.5 ± 9.0 years		150 mg: 12.3% 200 mg: 6.1%	Persistent ADAs were associated with lower sarilumab levels but no correlation with clinical efficacy	There was no evidence that ADA status was linked to adverse effects No notable differences in hypersensitivity reactions based on ADA status (no comparative statistics performed)
Genovese *et al*.^72^	Multicentre RCT of sarilumab	RA 150 mg: *n* = 400 50.1 ± 11.9 years 200 mg: *n* = 399 50.8 ± 11.8 years	150 mg: mean 9.5 years (range: 0.3–44.7) 200 mg: 8.6 years (0.3–34.2)		150 mg: 16.7% 200 mg: 13.0%	The presence of ADAs was not associated with discontinuations due to lack of efficacy	The presence of ADAs was not associated with hypersensitivity reactions
Xu *et al*.^73^	Worldwide Two-compartment model study of sarilumab	RA *n* = 1770 (F:M = 1466:304) Mean age: 52 ± 12 years	Data not available		18%	ADAs may be linked to higher drug clearance, but this study did not evaluate the impact on clinical efficacy	Data not available
IL-17 blockade (secukinumab/ixekizumab)							
Deodhar *et al*.^74^	Pooled clinical trial safety data for Secukinumab	PsA *n* = 1380 (F:M = 742:638) Mean age: 48.8 ± 12.0 years AS *n* = 794 (F:M = 265:529) Mean age: 42.4 ± 12.3 years	Data not available		<1% across all studies	No effect of ADA positivity on clinical efficacy was reported	Immunogenicity was not related to adverse effects
Mease *et al*.^75^	Multicentre phase III RCT of ixekizumab	PsA *N* = 417 (F:M = 225:192) Mean age: 49.5 ± 11.9	6.7 ± 7.2 years		5.3%	72.7% (8/11) of ADA+ve patients achieved a clinical response; no comparative statistics performed as very small sample size	Data not available
Gordon *et al*.^76^	Combined phase III RCTs of ixekizumab	Plaque psoriasis *n* = 1150	Data not available		9%	19 patients (1.7%) with high titres of ADAs had a lower clinical response than that of patients with no or low–moderate ADAs (no *p* value given)	Data not available
IL-12/23 blockade (ustekinumab)							
Strand *et al*.^28^	Systematic review	PsA Patient demographic data not available	Data not available		8–11% Concomitant use of MTX, AZA, leflunomide or mycophenolate is associated with lower rates of ADAs against INF in PsA	Data not available	Data not available
Smolen *et al*.^77^	Multicentre RCT	RA 90 mg/8 weeks *n* = 55 (F:M = 46:9) Age 50.8 ± 13.0 RA 90 mg/12 weeks *n* = 55 (F:M = 47:8) Age 51.1 ± 10.6	RA 90 mg/8 weeks 5.6 ± 5.5 RA 90 mg/12 weeks 6.8 ± 5.9		RA: 5.7% (3.3% neutralising)	Data not available	Data not available
IL-1 blockade (anakinra, canakinumab and rilonacept)							
Fleischmann *et al*.^78^	Multicentre RCT of anakinra	RA *n* = 1340 (F:M = 1045:354) Mean age: 55.2 years (range: 19–85)	10.3 years (range: 0.2–59.5 years)		50.1% (1.9% neutralising)	52% of those with neutralising ADA reported disease progression (no comparative statistics performed)	No associations between ADAs and adverse effects
Cohen *et al*.^79^	Multicentre RCT of anakinra	RA *n* = 419 Anakinra dose: 0.04 mg/kg/day *n* = 63 Mean age: 52.6 years 0.1 mg/kg/day *n* = 74 Mean age: 53.0 years 0.4 mg/kg/day *n* = 77 Mean age: 52.8 years 1.0 mg/kg/day *n* = 59 Mean age: 49.0 years 2.0 mg/kg/day *n* = 72 Mean age: 54.1 years	0.04 mg/kg/day: 6.3 years 0.1 mg/kg/day: 8.8 years 0.4 mg/kg/day: 7.0 years 1.0 mg/kg/day: 6.5 years 2.0 mg/kg/day: 8.0 years		2.7% (8 out of 297 screened for antibodies)	No impact on clinical efficacy was found	87.5% of ADA positive patients experienced injection-site reactions; no *p* value reported
Ilowite *et al*.^80^	Multicentre RCT of anakinra	JIA *n* = 25 (F:M = 17:8) Mean age: 10 years (range: 3–17)		Mean: 3.9 years (range: 1–11)	72% (none were neutralising)	No impact on clinical efficacy was found	Data not available
Sun *et al*.^81^	Prospective study of canakinumab	JIA *n* = 201 Age range: 2 to <20 years			3.1% (6 of the 14 patients screened for antibodies were positive, giving an incidence of 6/196)	No evidence of loss in clinical efficacy was found Observed trough canakinumab concentrations in ADA+ve patients were comparable with ADA−ve patients (no comparative statistics performed)	No association was demonstrated between ADAs and adverse effects
Ruperto *et al*.^82^	Multicentre RCT of canakinumab	JIA *n* = 50 (F:M = 28:22) Median age: 8.0 years (IQR: 6.0–12.0)		Median: 2.7 years (IQR: 1.3–6.2)	8% (4/50 patients) None were neutralising	Data not available	Data not available
Lovell *et al*.^83^	USA RCT of rilonacept	JIA *n* = 24 (F:M = 16:8) Mean age: 12.6 ± 4.3 years		3.1 years (mean)	54.2% (13/24)	No correlation between ADA and clinical responses was found Statistical testing not performed due to small sample size	All patients who experienced ⩾3 injection-site reactions were ADA+ve

+ ve, positive; −ve, negative; ADA, anti-drug antibody; ARDS, autoimmune rheumatic diseases; AS, ankylosing spondylitis; AZA, azathioprine; DMARD, disease-modifying antirheumatic drug; F, female; INF, infliximab; IL, interleukin; IQR, interquartile range; IV, intravenous; JIA, juvenile idiopathic arthritis; M, male; MTX, methotrexate; PsA, psoriatic arthritis; pSS, primary Sjögren syndrome; RA, rheumatoid arthritis; RCT, randomised control trial; RTX, rituximab; SC, subcutaneous; SD, standard deviation; SLE, systemic lupus erythematosus; TCZ, tocilizumab.

When anti-TNF-α agents have been studied comparatively in adults, there was evidence of increased prevalence of ADAs against infliximab compared with adalimumab (25.3% *versus* 14.1% respectively), as well as between adalimumab and golimumab (14.1% *versus* 3.8%).^25^ A similar trend was found in a meta-analysis of biologic agents in JIA, where the pooled prevalence of ADAs against infliximab was 36.6% compared with 21.8% for ADAs against adalimumab.^35^ As mentioned above, the prevalence of ADAs against golimumab seems to be higher in children (46.8%) but based on limited evidence.^39^

### Variable impact of ADAs directed against anti-TNF-α treatments on clinical efficacy: loss of efficacy to adalimumab and infliximab was consistently found in children and adults who developed ADAs

Various studies in RA, PsA and AS provided evidence for an association between the presence of ADA against adalimumab and loss of clinical efficacy or diminished clinical response,^23,28,31,50^ while other studies found no association^53,54^ (Table 1). The impact of ADAs on the trend of inflammatory markers is not clear; some studies found higher erythrocyte sedimentation rate (ESR) and C-reactive protein (CRP) in patients who had detectable ADAs,^27,31^ whereas other studies found no such association.^53^ In addition, the presence of both ADA and low adalimumab concentration at 3 months were together significant predictors of poor response at 12 months.^50,52^ However, the risk of flares following various adalimumab tapering strategies in RA did not seem to be influenced by the adalimumab serum levels or ADA prevalence.^84^

A higher proportion of ADA-positive JIA patients treated with adalimumab experienced loss of response and more clinical relapses than those without ADAs.^28,30^ In JIA, it was noted that transient ADAs (defined as measurable ADAs on up to two consecutive time points which disappeared on subsequent measurements without having any impact on treatment efficacy of toxicity) were not associated with diminished response to medication, whereas permanent ADAs did lower treatment response.^34^

Most adult rheumatology studies found no detectable ADAs against etanercept.^27,44^ It has been suggested that neither etanercept concentrations nor ADA positivity correlated with JIA activity or remission states.^37^

A meta-analysis of nine studies of infliximab in adult autoimmune diseases found that the presence of ADAs decreased the odds of response by 58%.^25^ After 52 weeks of treatment with infliximab, non-responder RA patients were significantly more likely to be ADA positive.^47^

Adult RA studies found that ADAs against golimumab were associated with a poorer clinical response.^28,56^ ADA-positive RA patients (15.2% at 24 weeks) had a worse EULAR response and higher DAS-28 compared with ADA-negative patients.^56^ However, one study which utilised a more sensitive method of ADA detection (drug-tolerant enzyme immunoassay, DT-EIA) in adults, reported no effects of ADAs to golimumab on clinical responses at 24 and 52 weeks, across RA, PsA and AS.^40^ This highlights the importance in sensitivities of assays used. Studies in children with JIA found that ADAs to golimumab did not appear to have impact on clinical responses.^39,57^ Brunner *et al*.^39^ reported that none of the eight JIA patients found with high ADA titres >1:1000, experienced flares.

ADAs against certolizumab appeared to have an impact on RA clinical response at 3 months, where the majority of ADA-positive patients were non-responders,^42^ but there was no independent correlation with the 12-month EULAR response,^43^ suggesting that there was a time-dependent relationship. There are no paediatric studies.

A meta-analysis performed on 12 observational prospective cohort studies in adults demonstrated that the development of ADA reduced the anti-TNF response rate (RR) by 68% [RR = 0.32; 95% confidence interval (CI) 0.22, 0.48],^85^ while in children with JIA, a qualitative analysis found that antibodies to infliximab and adalimumab were associated with treatment failure.^35^

### Additional methotrexate treatment decreased the rate of ADA formation against anti-TNF-α treatments

Generally, for both adults and children, concomitant DMARD therapy was beneficial and resulted in a decrease in ADA positivity, but the impact of DMARDs on ADA formation was not always analysed to enable reliable conclusions^9,30^ (Table 1). Most studies looked at concomitant methotrexate (MTX) therapy, but azathioprine, leflunomide and mycophenolate have also been shown to be associated with lower ADA prevalence, suggesting that all DMARDs may be associated with benefits against drug-induced immunogenicity.^23,28,32,52^ Unfortunately, none of the studies evaluated comparatively the impact of individual DMARDs on immunogenicity in inflammatory arthritis because of small numbers of patients on DMARDs other than MTX, and because some patients were treated with more than one conventional DMARD. Concomitant use of MTX was associated with lower rates of ADAs against infliximab in RA.^28,32,45,50,86^ Moreover, RA patients treated with infliximab were less likely to develop ADAs if they received high biologic doses/induction therapy, or if they received continuous *versus* intermittent therapy.^28,33,44,45,86^ A randomised controlled trial (RCT) of infliximab plus MTX for the treatment of JIA, found that more patients achieved clinical response in the ADA-negative group (79% *versus* 67%).^36^

Similar evidence has been found in children, with studies suggesting a protective effect with the addition of MTX.^34,35,57^ Interestingly, DMARD use in children was found to be significantly lower in those who developed permanent ADAs to adalimumab.^34^ It has also been suggested that MTX reduces immunogenicity against adalimumab in a dose-dependent manner,^44,50^ as patients who did not develop ADAs were on a higher MTX dose.^55^ However, a paediatric study found that there was no difference in ADA rates in JIA patients with longer exposure to MTX.^30^

In adults, concomitant use of MTX was associated with lower incidence of ADAs to golimumab.^28,40,87^ A study found that the mean trough golimumab level at 24 weeks was comparable in ADA-positive *versus* -negative patients, with or without concomitant MTX.^87^

### ADAs against infliximab and adalimumab have been associated with side effects to therapy

In both adults and children, there was no clear consensus on whether ADAs have an impact on safety (Table 1). As expected, most reports included a small number of cases experiencing side effects. Adverse events more frequently mentioned included injection-site or infusion reactions, serum sickness and thromboembolic events. Some studies suggested that adverse events occurred more frequently in patients with ADAs to adalimumab,^28,31,33^ with others showing no significant differences.^27,54^ In paediatric studies, despite limited information available, no association between the presence of ADA and adverse events was reported.^35^ There was a suggestion of a possible increase in minor upper respiratory tract infections in children with detectable ADAs; however, this conclusion was limited by the small sample size.^34^

ADAs against infliximab have been reported to confer a higher likelihood of adverse drug reactions.^25,28,33,44,45,48,50^ In an RA study,^48^ ADA-positive patients had an increased risk of adverse drug reactions compared with ADA-negative patients over 52 weeks [21 (18%) *versus* 7 (7%), *p* < 0.018].^50^ Similarly, JIA infusion reactions to infliximab were more commonly seen in ADA-positive patients (58% *versus* 19%).^36^ A retrospective chart review of children with JIA and paediatric inflammatory ocular diseases found that patients with ADAs had a 15-fold increased risk of infusion reactions to infliximab compared with patients without ADAs.^29^ This study also found that ADA-positive children were significantly younger (mean age 7.01 *versus* 9.88 years, *p* = 0.003).

Limited data were available regarding the impact of immunogenicity against etanercept on safety. Studies across age did not report an association between ADA positivity and adverse events.^35,57^ In JIA studies, the proportion of patients with ADAs did not differ between responders and non-responders to etanercept.^37^

Studies in both paediatric and adult populations did not report an association between ADAs and adverse effects to golimumab.^39,56,57^ Similarly, multiple adult studies reported no association between the presence of ADAs against certolizumab and adverse effects;^42,43,59^ in addition, RA patients who experienced adverse effects did not have ADAs.^42,43^

### Immunogenicity to anti-TNF-α biosimilars is similar to or lower than that of their originators

Biosimilars are new biological products which are highly similar to their biological reference drug and have comparable clinical efficacy. At present, the use of biosimilars in JIA is limited, thus most evidence related to their immunogenicity is available from adult studies. Multiple studies have shown similar clinical efficacy and immunogenicity profiles when comparing biosimilars with their reference products.^28,88–96^ For example, ADA-positive CT-P13 (an infliximab biosimilar) patients showed less clinical improvement.^28^ ADA against infliximab and adalimumab biosimilars were associated with lower drug concentrations.^93,97^ The PLANETRA study found that peak serum CT-P13 concentrations were reduced in the ADA-positive group (*C*_max_ = 85.1 µg/ml) compared with the ADA-negative subset (*C*_max_ = 96.7 µg/ml).^93^ One meta-analysis reported on the pooled response rates (RRs) of ADA against anti-TNF-α biosimilars compared with their reference product.^90^ There were no significant differences in ADA formation rates between the infliximab and adalimumab biosimilars and their reference drugs at 24 to 30 weeks. The etanercept biosimilars showed significantly lower rates of ADA formation compared with the reference product, with a pooled RR = 0.05 at 24–30 weeks.^90^ A study of etanercept biosimilar GP2015 did not detect any neutralising ADAs, and all ADA responses were transient (absent by week 24).^96^

## Clinical relevance of ADAs against other biologic agents in adult and paediatric inflammatory arthritis studies

### ADAs against abatacept are mainly non-neutralising and do not have significant impact on clinical efficacy unless treatment is temporarily discontinued

The prevalence of ADAs to fusion proteins, such as abatacept (which comprises an Fc region of IgG1 fused to the extracellular domain of CTLA-4) is generally acknowledged to be lower than to therapeutic mAbs. The prevalence of ADAs to abatacept ranged from 1% to 20% in adult studies,^28,44,51,65^ and from 8.7% to 23.3% in paediatric studies^35^ (Table 2). Younger children with JIA (2–5 years) had a higher prevalence of ADAs than older children (6–17 years).^63^ One JIA study compared the prevalence of abatacept specific ADA with anti-CTLA-4-specific antibodies and found the latter to be much higher (1.2% *versus* 20.7%).^97^ In terms of timing of the development of ADAs in children, one study found that ADA concentration increased with a longer duration of exposure to abatacept,^62^ whereas another found no increase with continued exposure.^64^

Similar to etanercept, abatacept generated ADAs which bind to the Fc fragment (hinge region) and have no neutralising activity.^28^ Non-neutralising ADAs decreased the circulating levels of abatacept by enhancing drug clearance in adults.^44,51^ In children, ADAs were also found to be non-neutralising but were not found to be associated with low abatacept concentrations.^62,97^

No loss of efficacy due to ADA against abatacept was found in JIA studies,^35,62,64,97^ while in contrast, in adults with RA, intermittent treatment discontinuation led to higher incidence of immunogenicity and loss of clinical response.^65^ It was observed that adult patients who discontinued the treatment temporarily had higher ADA rates than those on continuous treatment (7.4% *versus* 2.6% respectively).^44^ Similarly, ADAs were more frequent in children with JIA who interrupted treatment and had abatacept concentration below therapeutic levels, suggesting that higher treatment doses may be beneficial against immunogenicity.^97^

Some adult studies suggested that intravenous therapy was associated with less immunogenicity than subcutaneous administration,^28,98^ while other studies found no difference.^44^ In JIA, no difference was found between the two routes of administration.^35^

In RA, concomitant MTX therapy did not significantly affect immunogenicity.^65^ In paediatric studies, the impact of MTX has not been studied.^35^ Reassuringly, ADAs against abatacept were not associated with increased risk for injection site reactions, hypersensitivity or any other safety concerns,^35,62,65,97^ even when patients have been followed up to 7 years.^64^

### ADAs against B-cell-targeted therapies are dose dependent and have impact on clinical efficacy and risk of adverse reactions

Rituximab is a chimeric mAb against CD20. There have been no paediatric studies investigating the relevance of ADAs against rituximab. However, ADAs against rituximab have been reported in 0–21% of adult RA patients.^28^ Additionally, ADAs were found to be associated with a reduced treatment response and higher rates of treatment serious adverse events.^28,61^ Lower serum rituximab concentrations have been reported in ADA-positive patients compared with ADA-negative patients in RA.^60^ Moreover, the use of higher rituximab doses and induction therapy has been associated with a decreased incidence of ADAs in RA.^28^

A meta-analysis reported that the pooled RR of ADA formation for rituximab biosimilars was 0.86 at weeks 24–28.^91^ Of note, the pooled RR of neutralising ADA formation at the same time point was 1.16. Neutralising ADAs were also of a very low incidence at week 72 in the rituximab biosimilar CT-P10.^92^ Multiple studies have demonstrated a similar side-effect profile for biosimilars, as higher rates of infusion-related reactions were present in ADA-positive patients compared with ADA-negative patients^28,88,89,94,95^ (Table 2).

### Neutralising ADAs against tocilizumab has no clear impact on clinical efficacy and potential on side effects in adults, while there is a trend for clinical impact in children

Tocilizumab is a humanised mAb against the interleukin-6 receptor (IL-6R). Several studies have reported low ADA rates in RA patients.^28,66,67^ ADA positivity has been recorded in 1.5% and 1.2% of RA patients receiving intravenous and subcutaneous tocilizumab, respectively, with a high proportion of these being neutralising ADAs^68^ (Table 2). The rate of ADA formation has not been seen to significantly differ in tocilizumab monotherapy *versus* combination therapy with conventional synthetic DMARDs.^68^ No correlation has been found between ADA rates and adverse events or a reduced treatment efficacy in adults.^51,68^ Similarly, low levels of ADAs to tocilizumab have been reported in JIA patients, with a pooled prevalence of 2.3% across four studies.^35^ However, neutralising antibodies against tocilizumab in JIA have indeed been shown to correlate with treatment failure, as well as with infusion and hypersensitivity reactions.^35,69^ Yokota *et al.*^69^ found that out of five JIA patients treated with tocilizumab who developed ADA, four (80%) withdrew from the study due to infusion reactions.

### ADAs to sarilumab seem to have limited impact on clinical efficacy and no impact on adverse events

Sarilumab is human recombinant mAb that blocks both the soluble and membrane-bound IL-6 receptor, similarly to tocilizumab, but with a higher affinity. Currently, there are no studies of immunogenicity in paediatric populations. The presence of ADAs did not appear to affect clinical efficacy in various trials.^70–72^ The MONARCH trial demonstrated that only 2.7% of RA patients had persistent ADAs, however, no neutralising ADA were detected.^70^ It has been suggested that ADAs against sarilumab are, in most cases, transient.^99^ Xu *et al.*^73^ described a trend towards higher apparent linear clearance of sarilumab when ADAs were present. In addition, patients with persistent ADAs had a lower mean drug levels compared with ADA-negative patients. At a dose of 150 mg, treatment-emergent ADA incidence was 24.6% compared with 18.2% at a higher dose of 200 mg. Of those who had persistent ADA, the incidence of neutralising ADA was also higher in the group receiving 150 mg sarilumab compared with 200 mg (10.8% and 3.0% respectively).^71^ Multiple studies have shown that ADA positivity was not associated with a higher incidence of adverse effects.^70–72^ Hypersensitivity reactions occurring during treatment were reported in 8.0% of ADA-negative patients and in 3.1% of ADA-positive patients.^72^

### Neutralising ADAs against IL-12/23 blockade have low prevalence but possible impact on clinical efficacy in inflammatory arthritis

Ustekinumab is a human immunoglobulin G1κ mAb against common sub-unit p40 of IL-12 and IL-23. The prevalence of ADAs was 8% to 11% in psoriatic arthritis adult patients treated with ustekinumab.^28^ Moreover, a study evaluating the efficacy of subcutaneous ustekinumab in the treatment of RA reported that 7/123 (5.7%) of patients had ADAs, while 4/123 (3.3%) had neutralising ADAs.^77^ In this study, serum concentrations of ustekinumab were generally lower in ADA-positive patients^77^ (Table 2). There is evidence that neutralising ADAs against ustekinumab were associated with lower drug levels and loss of clinical efficacy in psoriasis and Crohn’s disease,^100,101^ suggesting overall that they may have similar impact in inflammatory arthritis. The relevance of ustekinumab immunogenicity is yet to be studied in children.

### Very low prevalence of ADAs against IL-17 blockade has been reported, and no impact on side effects or clinical efficacy

Secukinumab is a mAb targeting IL-17A. The treatment is not licensed for children. In a recent systematic review, the prevalence of ADAs against secukinumab was 0–1%.^28^ A study evaluated the prevalence of ADAs at 52 weeks in patients with psoriasis, PsA and AS treated with secukinumab and found it to be <1%; ADAs were not associated with loss of efficacy, changes in drug levels or adverse events.^74^

Ixekizumab is a humanised mAb which targets IL-17A used for the treatment of plaque psoriasis, PsA and AS. The prevalence of ADAs was 5.3%^75^ and 9%^76^ in adult patients with psoriasis and PsA, respectively, and they occurred within the first 12 weeks of treatment.^76^ ADAs were found to be non-neutralising and did not correlate with the rate of adverse reactions (Table 2). Patients with psoriasis or PsA who developed ADAs against ixekizumab had low and constant titres, which did not significantly impact clinical response. No data in children are available.

### ADAs against IL-1 blockade do not have significant impact on clinical efficacy or side effects

Anakinra is a recombinant a human IL-1 recombinant receptor antagonist initially trialled in RA, where it has been associated with a prevalence of ADA ranging from 50.1% to 70.9%.^78,79^ Similar to other recombinant proteins, only a small proportion of ADAs were neutralising (25/1240, 1.9%)^78^ (Table 2). Of these 25 RA patients, 13 (52%) reported disease progression; however, no relationships were found between neutralising antibody status and the occurrence of severe allergic reactions, malignancies, opportunistic infections, or serious infections.^78^ One study assessing the efficacy of anakinra in patients with JIA found that the prevalence of ADAs increased from 75% at 12 weeks to 82% at 12 months.^80^ At 12 weeks, all 4/64 (6%) of patients who had neutralising antibodies to anakinra were non-responders to treatment.^80^ However, non-neutralising antibodies to anakinra were not associated with a reduced response to treatment.^80^ There have been no studies analysing the association between ADAs to anakinra and adverse events in JIA.

Canakinumab is a fully human mAb against anti-IL-1β used in systemic-onset JIA (soJIA). Studies in children with systemic JIA found a prevalence of ADAs against canakinumab of 3.1% (6/196),^81^ and 8%,^82^ and ADAs had no neutralising capacity and did not affect the drug levels or the rate of side effects.

Rilonacept is a fully human dimeric fusion protein that acts as a soluble decoy receptor which blocks IL-1β. An RCT in soJIA did not find an association between ADA positivity and clinical response.^83^ This trial found that 54.2% (13/24) of patients developed ADA during the 23-month period of open-label treatment (following a 4-week double-blind treatment phase). There was no correlation between ADA positivity and plasma levels of rilonacept.^83^ Although the sample size was small, this study noted that the patients who developed at least three injection-site reactions were all ADA positive, thus suggesting there is an association between ADAs and adverse effects.

## Conclusion

Immunogenicity to biologic treatment has been investigated in various types of inflammatory arthritis in children and adults. The overall impression is that immunogenicity to biologics used in rheumatology was not particularly confounded by clinical indication or significantly affected by patients’ age (Table 3). However, a direct comparison between the studies evaluated by this report is not possible, because of the high study heterogeneity, a low number of studies investigating less commonly used biologic treatments and high variability between the methods of ADA detection and time points of ADA measurements, study design and concomitant MTX therapy.

**Table 3.** Comparison between the prevalence ranges for ADAs to various biologic agents in adult *versus* paediatric populations.

Prevalence of ADAs	Adults with inflammatory arthritis (%)	Children with juvenile idiopathic arthritis (%)
TNF-α blockers		
Adalimumab and biosimilars	0–67	6–45
Infliximab and biosimilars	6.1–62	26–37
Etanercept and biosimilars	0–13	0–33
Golimumab	2–39.9	46.8
Certolizumab	2.8–65	Data not available
B-cell depletion		
Rituximab and biosimilars	0–21	Data not available
Co-stimulatory blockade		
Abatacept IV	2–20	2–11
Abatacept SC	2–20	2–11
IL-6 blockade		
Tocilizumab	0–16	1–8
Sarilumab	7–24.6	Data not available
IL-17 blockade		
Sekukinumab	0–1	Data not available
Ixekizumab	5.3–9	Data not available
IL-12/23 blockade		
Ustekinumab	5.7–11	Data not available
IL-1 blockade		
Anakinra	50.1–70.9	81.8
Canakinumab	Data not available	3.1–8
Rinolacept	Data not available	54.2

ADA, anti-drug antibody; IL, interleukin; IV, intravenous; SC, subcutaneous; SD, standard deviation; TNF, tumour necrosis factor.

As there are some differences between the biologic agents approved for use in paediatric *versus* adult rheumatic diseases, in some cases there were no data available to enable comparisons between the two populations (e.g. certolizumab, sarilumab, secukinumab, ustekinumab and ixekizumab have no studies in children, while rilonacept and canakinumab are not commonly used in adults). The discrepancy found between the rate of ADAs against golimumab is not easy to interpret because they have been investigated only in one study in JIA.

This literature review provided evidence for variable prevalence of ADAs depending on the study methodology, sample size, time points for sample evaluation, concomitant DMARD therapy, as well as laboratory assays used for ADA detection. Overall, the highest ADA prevalence was found in patients treated with mAbs against TNF-α and recombinant human IL-1 receptor antagonist (anakinra), although the impact of ADAs on clinical efficacy was clearly influenced by their neutralising properties and impact on drug levels. In contrast to immunogenicity to IL-1 blockade, which had minimal or no impact on clinical efficacy as the proportion of neutralising ADA was very low, ADA against adalimumab, infliximab, certolizumab, and to a certain extent, golimumab had a significant impact on clinical efficacy. As a consequence, the choice of biologic therapeutic agent for individual patients influences their immunogenicity monitoring strategy.

All mAbs against TNF-α (and their biosimilars) were associated with higher prevalence of ADAs than etanercept (a fusion protein) and this is probably explained by the structure of the biologic agent as well as frequency of administration, which in the case of etanercept, ensures more constant serum drug levels. It is recognised that anti-idiotypic ADAs against therapeutic mAbs usually target the drug-binding site, as this does not belong to the patient immunoglobulin repertoire, therefore these ADAs have neutralising properties with impact of drug efficacy and they are clinically relevant.^33^ The detection of neutralising ADAs in certain patients should be monitored and correlated with clinical response and drug levels to guide further therapeutic decisions.^102^ Neutralising ADAs have been found in patients treated with adalimumab, infliximab, certolizumab pegol and golimumab, as well as tocilizumab, ustekinumab and secukinumab.

By contrast, in the case of fusion proteins which comprise a naturally occurring receptor fused with the constant region of human Ig, the immunogenicity process is primarily triggered by the recognition of the fusion part of the molecule with no direct impact on the drug-binding site. Overall, these therapeutic agents were associated with less immunogenicity, although neutralising ADAs against fusion proteins have also been described with both etanercept and abatacept,^65,103^ suggesting that their monitoring could be relevant in selected categories of patients, especially if the treatment has been discontinued temporarily.

Despite the potential side effects associated with the presence of ADAs overall, irrespective of their neutralising properties, detection of ADAs does not preclude loss of clinical response, as long as it does not reduce the serum concentration of the biologic agent below the therapeutic threshold,^33^ therefore monitoring of ADA without drug levels has no clinical relevance.

High ADA concentration correlated with lower drug levels and impact on clinical efficacy when patients of all ages were treated with adalimumab, infliximab, golimumab, certolizumab, rituximab, abatacept, anakinra, canakinumab, and possibly ustekinumab, while the presence of ADA had less impact on clinical efficacy in adult patients treated with IL-6 and IL-17 blockage and children treated with rilonacept (IL-1β decoy receptor). Patients with higher ADA titres and lower or not/detectable drug levels are probably at risk of losing clinical efficacy and need to be monitored more closely.

It is clinically important to take into consideration the fact that not all detectable neutralising ADAs had impact on clinical outcomes (e.g. tocilizumab ADAs lowered treatment response in children with JIA but less in adults with RA). Neutralising ADAs were more commonly found in patients treated with mAbs compared with fusion proteins; however, not all ADAs against mAbs had neutralising properties or impact on clinical efficacy (e.g. ADAs against ixekizumab were predominantly non-neutralising and did not influence clinical response).

The timing of developing ADAs varied according to the type of biologic treatment and patients’ age. Patients developed ADAs against adalimumab earlier in their disease course, while ADAs in children with JIA treated with abatacept increased with longer time exposure to the drug.

Although data from paediatric studies are scarce overall, studies found that younger age in children with JIA was associated with a higher prevalence of ADAs, as well as side effects to certain biologics, suggesting that caution in monitoring younger patients is advisable.

There is good evidence that higher doses of rituximab and infliximab, as well as more regular administration (as in the case of etanercept) were associated with lower ADA prevalence, suggesting that medication discontinuation and tapering biologic treatment doses could have impact on clinical efficacy. Monitoring patients’ compliance and taking into consideration their dosing regimen, route and frequency of biologic medication administration are important aspects of immunogenicity risk assessment. Increasing treatment dose as well as switching to intravenous formulations can lower the ADAs and restore treatment response; therefore, these are useful therapeutic strategies to address the clinical impact of drug-induced immunogenicity.

In addition, the large variability of ADA levels against biologic agents detected in various adult and paediatric studies of inflammatory arthritis is very likely influenced by the sensitivity of the assay used, concomitant MTX dose, time point of sample collection, as well as patients’ characteristics (genetic background, smoking, age). The overall impact of ADAs on drug efficacy, as well as therapeutic drug monitoring, are particularly relevant in guiding future therapeutic strategies of tapering biologic treatments in inflammatory arthritis patients,^102,104^ although further research related to their impact on clinical decision making is required.^16,84^

Based on data available in the literature, concomitant treatment with MTX to address the risk of immunogenicity is recommended in patients treated with abatacept, infliximab, golimumab, while in the case of treatment with etanercept, abatacept and tocilizumab, the impact of additional MTX is not significant.

**Figure 1.**
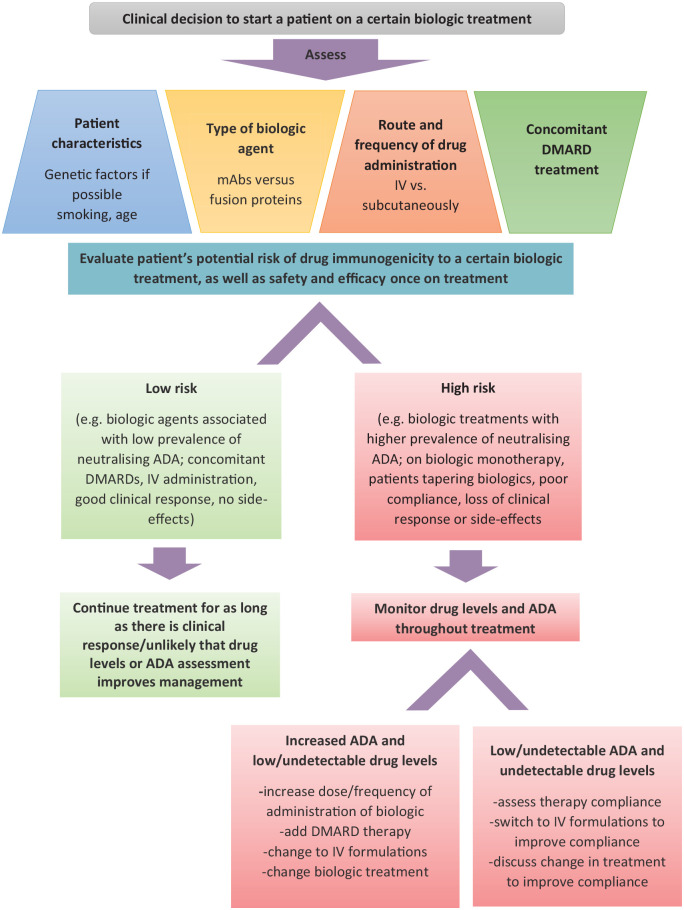
Potential clinical applications of the assessment of immunogenicity to biologic treatments. ADA, anti-drug antibody; IV, intravenous; DMARD, drug-modifying antirheumatic drug; mAbs, monoclonal antibodies.

We propose a potential strategy for drug immunogenicity monitoring for improved clinical benefit (Figure 1). The main clinical instances when ADAs and drug levels should be monitored is loss of clinical efficacy, monotherapy with biologic agents recommended to be prescribed in addition to MTX, clinical reasons for frequent dose intermittent discontinuation, in patients who tapered biologics (especially administered subcutaneously), patients who develop infusion/injection reactions and other side effects to therapy. Further research especially focused on patient individual risk to develop immunogenicity to biologics is required to enable personalised therapy selection.
